# Histological assessment of optogenetic tools to study fronto-visual and fronto-parietal cortical networks in the rhesus macaque

**DOI:** 10.1038/s41598-020-67752-6

**Published:** 2020-07-06

**Authors:** Michal G. Fortuna, Janina Hüer, Hao Guo, Jens Gruber, Eva Gruber-Dujardin, Jochen F. Staiger, Hansjörg Scherberger, Stefan Treue, Alexander Gail

**Affiliations:** 1 Cognitive Neuroscience Laboratory, German Primate Center - Leibniz Institute for Primate Research, 37077 Göttingen, Germany; 2 Primate Genetics Laboratory, German Primate Center - Leibniz Institute for Primate Research, 37077 Göttingen, Germany; 3 Pathology Unit, German Primate Center - Leibniz Institute for Primate Research, 37077 Göttingen, Germany; 4 Institute for Neuroanatomy, University Medical Center, University of Göttingen, 37075 Göttingen, Germany; 5 Neurobiology Laboratory, German Primate Center - Leibniz Institute for Primate Research, 37077 Göttingen, Germany; 6 Bernstein Center for Computational Neuroscience, 37077 Göttingen, Germany; 7 Faculty of Biology and Psychology, University of Göttingen, 37077 Göttingen, Germany

**Keywords:** Neuroscience, Physiology

## Abstract

Optogenetics offers unprecedented possibilities to investigate cortical networks. Yet, the number of successful optogenetic applications in non-human primates is still low, and the consequences of opsin expression in the primate brain are not well documented. We assessed histologically if we can target cerebrocortical networks with three common optogenetic constructs (AAV2/5-CaMKIIα-eNpHR3.0-mCherry, -ChR2-eYFP, -C1V1-mCherry). The frontal eye field or the dorsal premotor area of rhesus macaques were virally injected, and the resulting transduction spread, expression specificity, and opsin trafficking into axons projecting to parietal and visual areas were examined. After variable periods (2–24 months), expression was robust for all constructs at the injection sites. The CaMKIIα promoter driven-expression was predominant, but not exclusive, in excitatory neurons. In the case of eNpHR3.0-mCherry and ChR2-eYFP, opsins were present in axonal projections to target areas, in which sparse, retrogradely transduced neurons could also be found. Finally, the intracellular distribution of opsins differed: ChR2-eYFP had almost exclusive membrane localization, while eNpHR3.0-mCherry and C1V1-mCherry showed additional intracellular accumulations, which might affect neuronal survival in the long-term. Results indicate that all three constructs can be used for local neuronal modulation, but axonal stimulation and long-term use require additional considerations of construct selection and verification.

## Introduction

Optogenetics, a method to selectively manipulate neuronal cell activity with light, has transformed the field of neuroscience by enabling experimental approaches that previously were either not possible or particularly challenging to accomplish^1–6^. In recent years, optogenetics has also been adapted for the use in non-human primates (NHPs)^7–11^, where the lack of transgenic animals hampered the use of otherwise widely utilized approaches to investigate neural circuits (e.g., gene knock-outs, inducible transgene expression, calcium sensors, etc.).

One of the most useful applications of optogenetics in systems neuroscience is the possibility to investigate causal interactions between brain areas. Neural interactions can be probed by activating or inhibiting genetically modified groups of cells by illuminating their cell bodies. This allows investigation of the role of these cells in the context of a wider neural network. Alternatively, one can manipulate specific efferent fibers in their termination zone, thereby determining the impact of a particular neural pathway^2,12^. So far, most optogenetic studies in NHPs have focused on perturbing defined brain areas by illuminating cell bodies within the injected area^10^. Alternatively, the prospect of selectively stimulating the axons of projection neurons of area A at their termination zone in a non-injected area B offers a unique way to establish causal interactions that underlie cognitive processes in behaving NHPs^13^. These two approaches probe different aspects of the neural circuitry and therefore address distinct questions, as shown in rodents^14,15^ and NHPs (see^16^ vs.^17,18^). Carefully choosing the most appropriate approach allows for a comprehensive understanding of causal interactions.

A successful optogenetic experiment requires a robust expression of the opsin protein in cells of interest, which in turn depends on the viral vector (lentivirus or adeno-associated virus—AAV, serotype, etc.), genetic promoter, expression cassette, and the delivery method^11,19–21^. Crucially, the opsin density on the cell membrane needs to be high enough for the light stimulation to cause sufficient de- or hyperpolarization of the cell. However, the optimal viral vector and the targeting strategy may vary between species, brain regions, and specific experimental needs, thus appropriate testing and post-experimental histological verification are important. While such verifications are routinely done in rodents, in NHPs such information is often unobtainable in time, due to the long-term use of experimental animals.

Another hurdle in the effective use of optogenetics in NHPs is their much larger brain size, making it more challenging to reliably transduce a large, yet delineated cortical volume. Furthermore, data collection periods typically last for many months, sometimes over a year, during which a stable (or predictable) opsin expression is desired. Two studies reported stable opsin expression upto 1–2 years after viral vector delivery^8,22^, but an occurrence of a humoral immune response following AAV1 and AAV9 virus injections was reported by others^23^. Overall, long-term stability of opsin expression or potential immune reactions has not been investigated sufficiently in the primate brain. Finally, the specificity of Ca2+/calmodulin-dependent kinase 2 alpha (CaMKIIα) promoter-driven expression in non-human primates is not rigorously established, and studies reported ectopic expression in non-glutamatergic neurons, including inhibitory GABAergic neurons, e.g., parvalbumin (PV) positive cells^20^.

To address these open questions pertinent to successful applications of optogenetics in NHPs, we tested a protocol for an area-specific, AAV2/5-mediated neuronal transduction in rhesus macaques and analyzed the resulting expression in terms of transduction spread/efficiency, neuronal specificity, and opsin expression in long-range axons at different time points post-injection (2, 4 and 24 months). Two most widely used excitatory opsins, i.e., the depolarizing Channelrhodopsin-2 (ChR2) and red-shifted chimera C1V1, and one inhibitory opsin (the enhanced version of hyperpolarizing halorhodopsin (eNpHR3.0)) were tested. Given our research interests^24–27^, we targeted two frontal lobe structures involved in visually guided behavior and attention, namely the dorsal premotor cortex (PMd) and the frontal eye field (FEF), and parietal and temporal areas receiving their projections, with the prospect of optical stimulation of both (injection and projection sites) during electrophysiological recordings in behaving NHPs.

## Results

**Figure 1:**
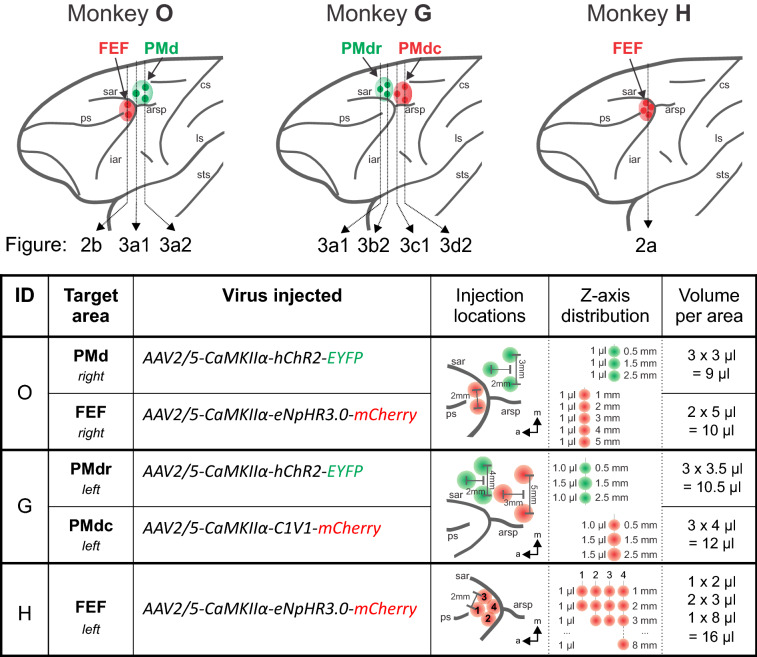
Localization of the injection sites and summary of the virus injection procedure. A schematic representation of a rhesus macaque brain with indicated injection sites in monkeys O, G, and H. Color-coded dots represent individual penetration sites in areas FEF and PMd. The same color code is used in the table, which summarizes viral vectors used and the injection arrangement at the target sites. Spatial distributions are sketched relative to anatomical landmarks (sar, arsp, and ps), and the distances between individual injection entry points are depicted. Virus distribution along the cortical depth (Z-axis distribution) and the total volume injected per target area are specified. Vertical dotted lines in the brain schematic mark approximate locations of the corresponding coronal sections shown in Figs. 2, 3; arrows and labels refer to corresponding panels in Figs. 2, 3. *sar* arcuate sulcus upper limb, *arsp* spur of the arcuate sulcus, *ps* principal sulcus, *cs* central sulcus, *iar* arcuate sulcus lower limb, *ls* lateral sulcus, *sts* superior temporal sulcus.

Three opsins were tested: depolarizing hChR2(H134R) and C1V1(E122T/E162T), and inhibitory eNpHR3.0 (see Methods and Fig. 1 for details). All opsin genes were fused with a fluorescent marker (ChR2 with enhanced yellow fluorescent protein—eYFP and eNpHR3.0 and C1V1 with monomeric red fluorescent protein—mCherry), expression was driven by the CaMKIIα promoter, and all constructs were packaged into AAV2/5 vectors. Two frontal lobe regions were virally injected: the PMd (monkey G and O) and the FEF (monkey H and O) (Fig. 1; see Methods for details of the protocol). After variable survival periods (monkey O: 2 months, G: 4.5 months, and H: 2 years), histological analyses were carried out.

### Expression at the injection sites

**Figure 2:**
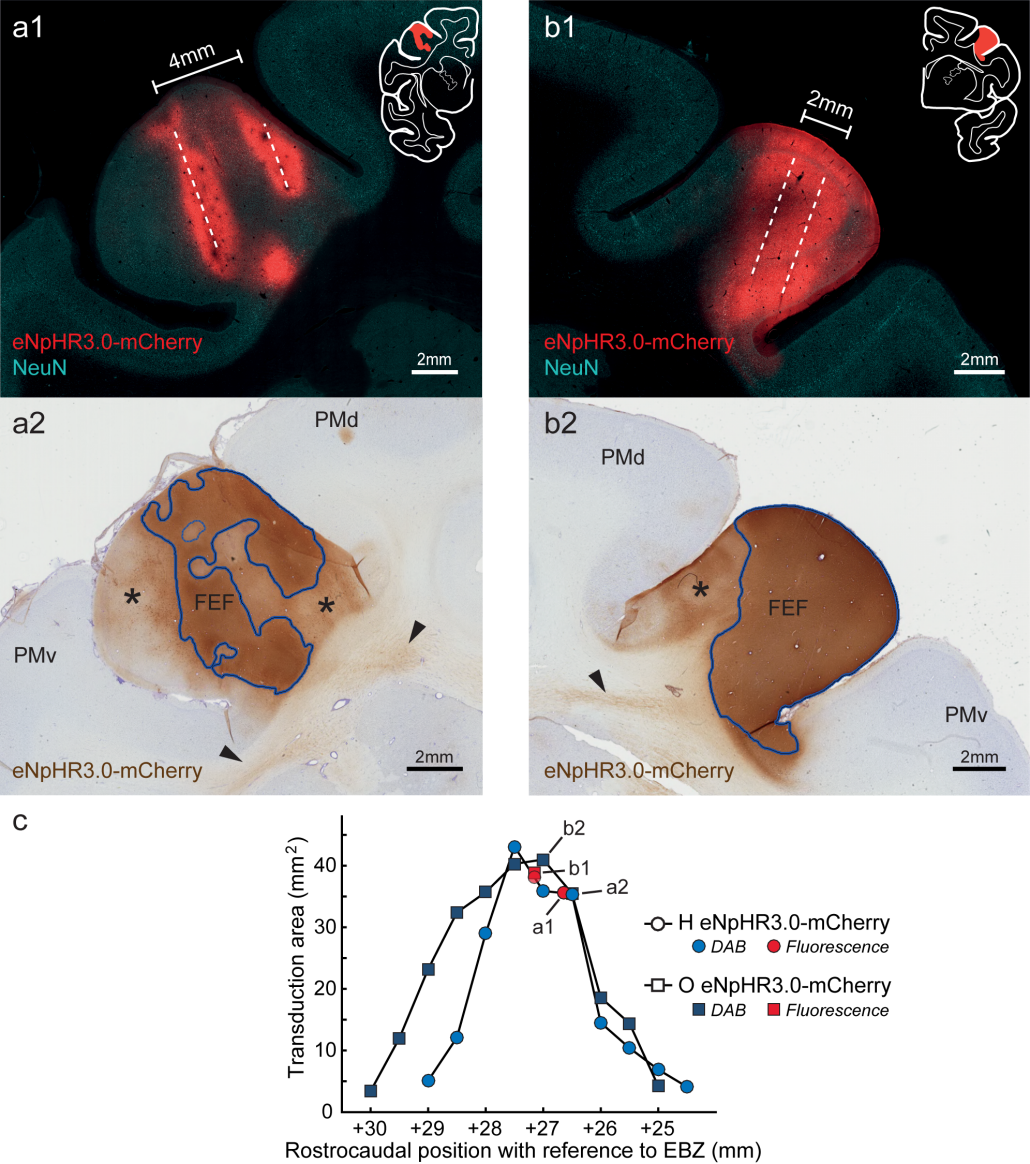
Expression of eNpHR3.0-mCherry in area FEF in monkey H (**a**) and monkey O (**b**), and resulting transduction spread. (**a1**, **b1**) Representative 50 µm coronal sections of the injected area FEF revealed by mCherry immunofluorescence. The outline of the complete coronal hemisection is shown for reference as inset. Dashed lines mark estimated injection tracks. (**a2**, **b2**) DAB stained coronal sections (150 µm apart from (**a1**) and (**b1**) respectively). Areas of dark staining (i.e., high opsin density) are outlined in blue, areas of lighter staining (i.e., lower opsin density) are marked with asterisks (and not included into transduction area measurement). Arrowheads point to labeled axons in the white matter leaving area FEF. (**c**) Transduction area measured in mm^2^ in the coronal plane and aligned rostrocaudally with the atlas coordinate system (+ mm with reference to Ear Bar Zero (EBZ), following the coordinates of the brain atlas by Saleem and Logothetis^63^). The transduction area was determined based on the dark stained areas indicated in (**a2**) and (**b2**) (see Methods for details). Fluorescent images were captured with an Axio Observer 7 epifluorescent microscope controlled by ZEN 2.3 (Zeiss), and DAB images were acquired with Aperio CS2 scanner (Leica); see Methods for details. *PMv* ventral premotor cortex.

**Figure 3:**
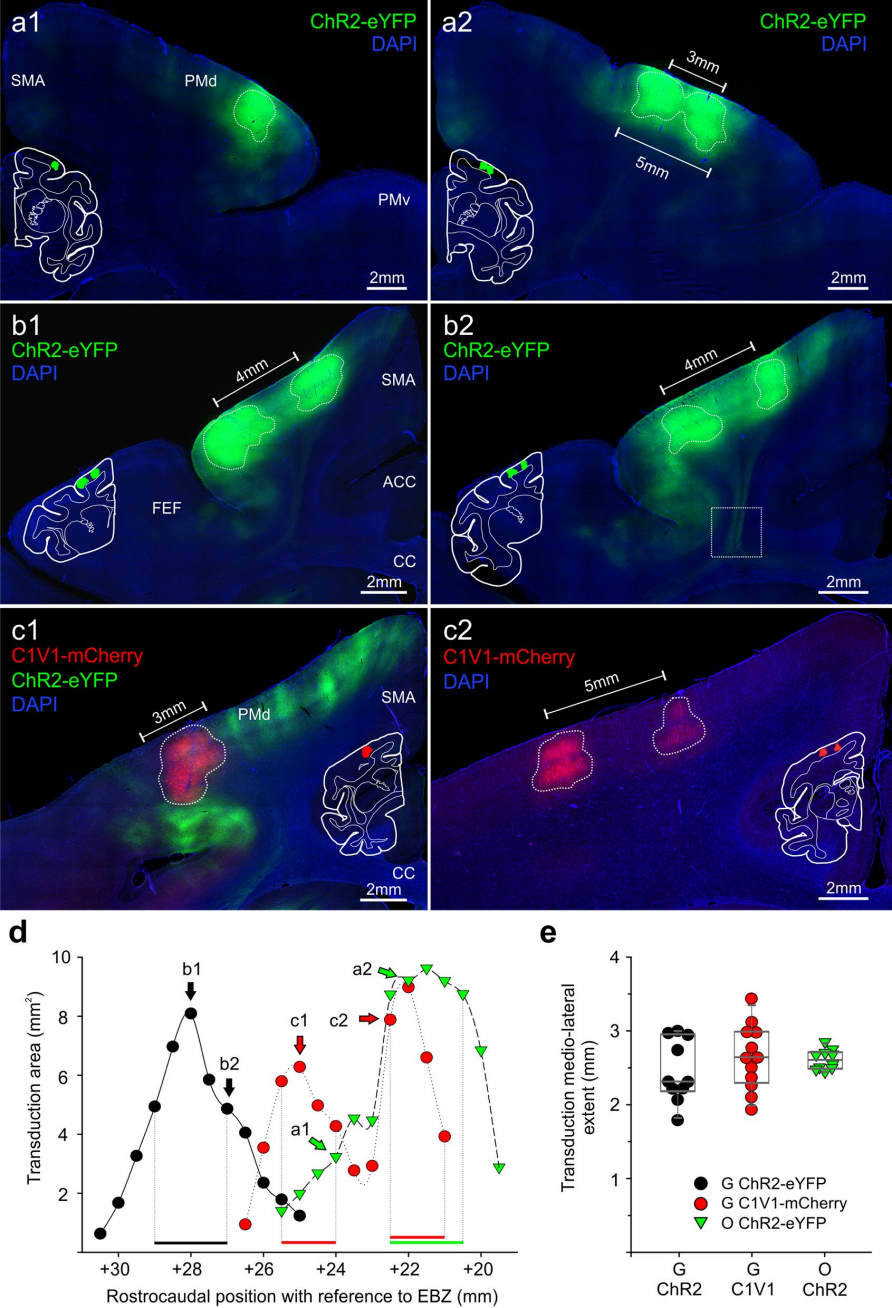
Transduction spread resulting from ChR2-eYFP and C1V1-mCherry PMd injections in monkey O (**a**) and monkey G (**b**,**c**). (**a**–**c**) Representative 50 µm coronal sections from area PMd, immunoreacted against eYFP (**a**,**b**) or mCherry (**c**), counterstained with DAPI. Corresponding, white-contoured hemisection with colored transduction regions are shown as a reference. Rostral (**a1**), and more caudally (**a2**) ChR2-eYFP transduced areas in monkey O; note that injection separation by 3 mm resulted in a virtual fusion of the two transduction zones in (**a2**). (**b1**, **b2**) Examples of the rostral aspect of PMd in monkey G, transduced with ChR2-eYFP; separation of the injections by 4 mm produced two separate transduction zones with a fluorescent area consisting of labeled processes and sparsely transduced neurons in between. In (**b2**), axons leaving area PMd can be seen (enlarged view in Fig. 5c). (**c1**, **c2**) Example images of the caudal aspect of PMd in monkey G, transduced with C1V1-mCherry. In c1, adjacent to mCherry-expressing neurons, eYFP^+^ projections from rostrally transduced cells (green-colored) can also be seen. (**c2**) shows two separate transduction areas, resulting from 5 mm mediolateral separation of the injection points. Note the lack of fluorescent processes in between, in contrast to ChR2-eYFP expressing areas. (**d**) Transduction area measured in the coronal plane and aligned to a rostrocaudal position (+ mm EBZ; as in Fig. 2c). Horizontal bars demarcate a 1.5–2 mm ‘core of the transduction’ for each case (note: C1V1-mCherry has a bimodal distribution due to wider rostrocaudal separation of the injection sites, therefore two core areas); the measure of the mediolateral extent of the transduction is derived from these areas, and plotted in (**e**). (**e**) illustrates individual values (dots), a median, and 25/75 percentiles (grey box) of the extent of the transduced area at the ‘core of the transduction’ (horizontal bars in (**d**) show which points were considered). Median values were not statistically different between groups (*P* = 0.519, 1-way analysis of variance on ranks). Images acquired with Axio Observer 7 controlled by ZEN 2.3 (Zeiss). *SMA* supplementary motor area, *PMv* ventral premotor cortex, *ACC* anterior cingulate cortex, *CC* corpus callosum.

The first question we wanted to answer was how effective the injection protocol was in transducing defined cortical structures, that is, how far the transduction had spread and how homogeneous the expression was between injection points. In all cases (three animals, five locations), virus injections resulted in strong transgene expression at the expected locations in FEF (Fig. 2) and PMd (Fig. 3).

**Figure 4:**
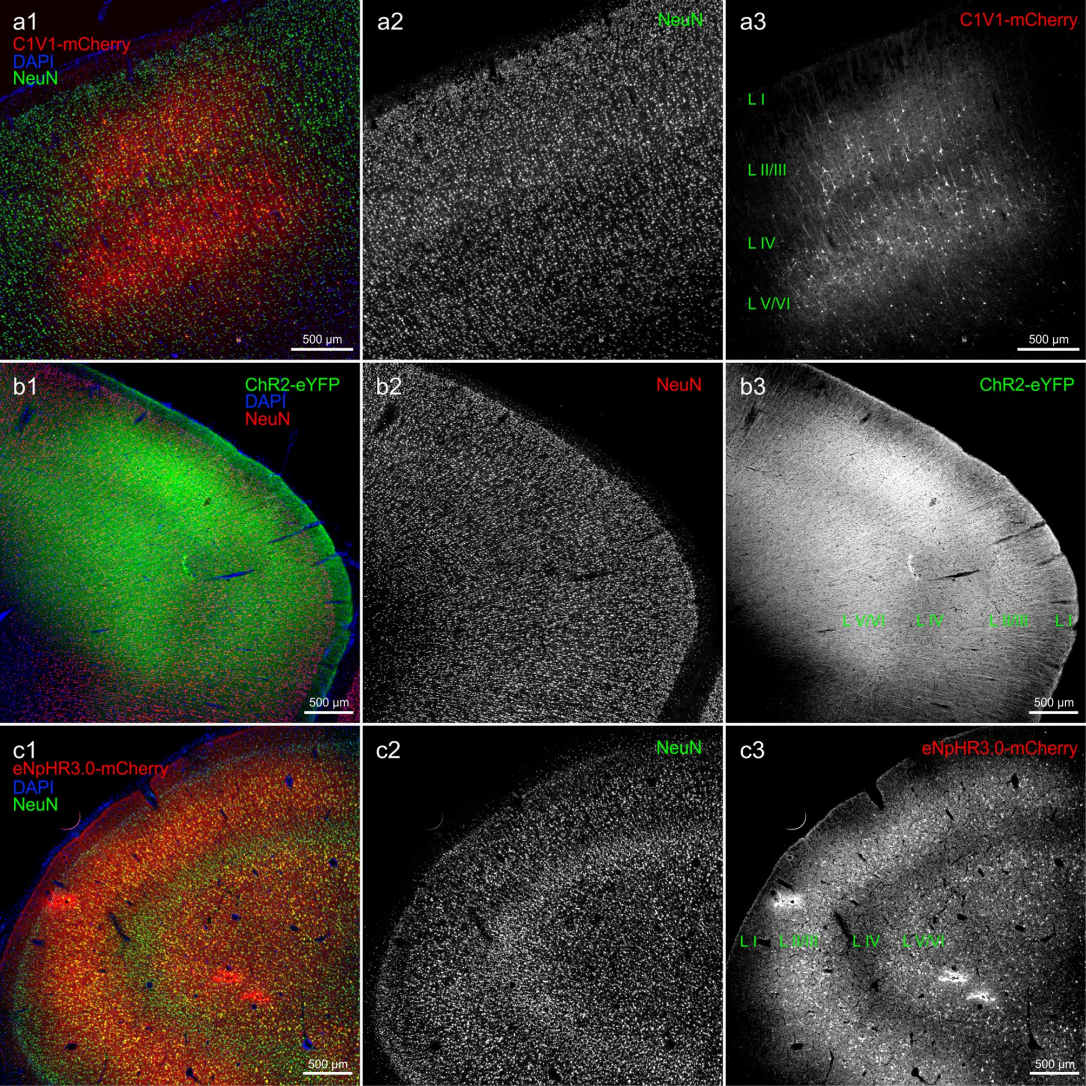
Comparison of ChR2-eYFP, C1V1-mCherry and eNpHR3.0-mCherry expression at the injection sites. (**a**) Low magnification images of area PMd transduced with C1V1-mCherry virus (monkey G); (**a1**) overlay image where neurons were identified by NeuN immunoreactivity ((**a2**); visualized with Alexa 647 and pseudocolored in green) and mCherry signal was amplified with Cy3 (**a3**); sections were counterstained with DAPI (blue). Note that cell bodies and immediate processes are predominately labeled, also a much lower density of transduced cells in putative layer IV. (**b**) Low magnification images of the PMd area transduced with ChR2-eYFP virus (monkey O); (**b1**) composite image where neurons were identified by NeuN immunoreactivity ((**b2**); visualized with Alexa 647 and pseudocolored in red) and eYFP signal was amplified with Alexa 488 (**b3**); sections were counterstained with DAPI. Note ubiquitous axonal and dendritic processes spanning through all layers of the cortex (including layer I) as opposed to much more restricted C1V1-mCherry expression. (**c**) Low magnification images of area FEF in monkey O transduced with the eNpHR3.0-mCherry virus (monkey O); (**c1**) composite image where neurons were detected with NeuN ((**c2**); visualized with Alexa 647 and pseudocolored in green) and mCherry signal was amplified with Cy3 (**c3**); sections were counterstained with DAPI. Note widespread opsin expression in cell bodies and their processes throughout the cortex, with an apparent lower density of labeled neurons in cortical layer IV. Images acquired with Axio Observer 7 controlled by ZEN 2.3 (Zeiss).

Injections into FEF (monkey H and O) resulted in robust expression of the eNpHR3.0-mCherry protein in cells around the injection tracks (Figs. 2, 4c). Although two different volumes were delivered in both monkeys (monkey O: 10 μl; monkey H: 16 μl), the injections in each animal produced a maximum area of around 40 mm^2^ at AP+27/ + 27.5 mm (coronal view; determined based on DAB staining), with a comparable rostrocaudal spread of about 4 mm (Fig. 2c).

Nonetheless, due to their localization, injections transduced slightly different parts of FEF in the two animals. In monkey O, transduction was most prominent in the dorsal and ventral subdivision of FEF (area 8Av, area 45), with noticeable expression in the ventrolateral prefrontal area (in the lower limb of the arcuate sulcus). Transduction in monkey H, instead, was higher in the dorsomedial part of FEF (area 8Ad), including aspects of the dorsolateral prefrontal area (in the upper limb of the arcuate sulcus). In monkey O, the virus was injected along two separate needle penetrations, approximately 2 mm apart. As shown in Fig. 2b1, this horizontal separation led to a homogenous expression in between, and about 1–1.5 mm around the needle tracts over the entire length of the penetration. In monkey H, which was analyzed 2 years post-injection, the transduced area appeared less homogenous than in monkey O. Despite the higher injected volume, the overall extent of the transduction seemed slightly smaller (Fig. 2a,c). As we were not able to unequivocally identify all four injection tracks, Fig. 2a1 illustrates expression areas around two likely penetrations, separated by ~ 4 mm, and resulted in two non-overlapping columns of opsin expressing cells (~ 2 mm in diameter each).

Conservatively, we included only intense, and rather uniformly transduced areas, adjacent to the potential needle tracks (Fig. 2a2, b2, blue outlines), and excluded regions of lower opsin density and inhomogeneous expression pattern (input fibers, extended dendrites, and spare cell groups; Fig. 2a2, b2, asterisks). Indeed, when also considering areas of lower opsin density, almost the whole anatomically defined area FEF showed some degree of expression.

Injections into PMd in monkeys G and O produced fairly homogeneous opsin expressions along the cortical depth, as well as in the mediolateral axis (Fig. 3). The ChR2-eYFP virus injections in PMd in monkeys G and O (Fig. 3a1, a2, b1, b2) yielded a seemingly broader extent of transduction than the one produced by the C1V1-mCherry injection in monkey G (for instance compare b2 with c2 in Fig. 3). This impression can be attributed to extensive dendritic and axonal expression of the ChR2-eYFP protein, leading to an omnipresent fluorescent signal at the injection sites, and intensely labeled processes and individual cells surrounding the main transduction zones (e.g. Fig. 3a1, b1). At the site of injection, labeled cell bodies were poorly distinguishable from the surrounding fluorescent neuropil (the subject of membrane expression is discussed in more detail below) (Fig. 4b). Nonetheless, homogeneous and restricted expression sites were identified, outlined, and the coverage estimated (Fig. 3—dotted outlines). This general appearance of ChR2-eYFP expression stands in sharp contrast to the C1V1-mCherry expression in PMd neurons; while being robust, the mCherry fluorescence was much more confined to cell bodies and their immediate processes (Fig. 3c1 and c2, see also Fig. 4a).

The resulting size of the ChR2-eYFP and C1V1-mCherry transduction area and its rostrocaudal distribution are shown in Fig. 3d. The ChR2-eYFP virus injections (monkey O and G), for which penetration tracks were separated by 2 mm rostrocaudally produced a bell-shaped area of transduction, with an effective spread of ~ 4 mm along this axis (Fig. 3d). The C1V1-mCherry injections were separated by 3 mm rostrocaudally, and resulted in broader (~ 5 mm), and bimodal distribution of transduction spread along the rostrocaudal axis (Fig. 3d). In all, PMd injections produced a maximum transduction area of ~ 10mm^2^ (per slice, in coronal view), which roughly corresponds to two neighboring opsin expressing areas, each measuring 2 mm in depth and 2.5 mm in diameter.

The mediolateral extent of effective transduction was estimated based on measures at the center and the surrounding of injection location, as indicated by horizontal bars in panel 3d (Fig. 3e). The average spread for all three cases was similar (no significant difference): Monkey G, ChR2-eYFP—2.31 mm, Monkey G, C1V1—2.64 mm, Monkey O, ChR2-eYFP—2.61 mm, ranging from 1.79 to 3.44 mm. This result agrees well with the rostrocaudal extent of the expression and indicates that the used approach of distributing 3–4 µl of a virus evenly along the cortical depth, results in a cortex-spanning cylindrical expression volume with an approximate radius of ~ 1–1.5 mm around each penetration site in both PMd and FEF.

### Cell-type specificity and cellular distribution of expression

**Figure 5:**
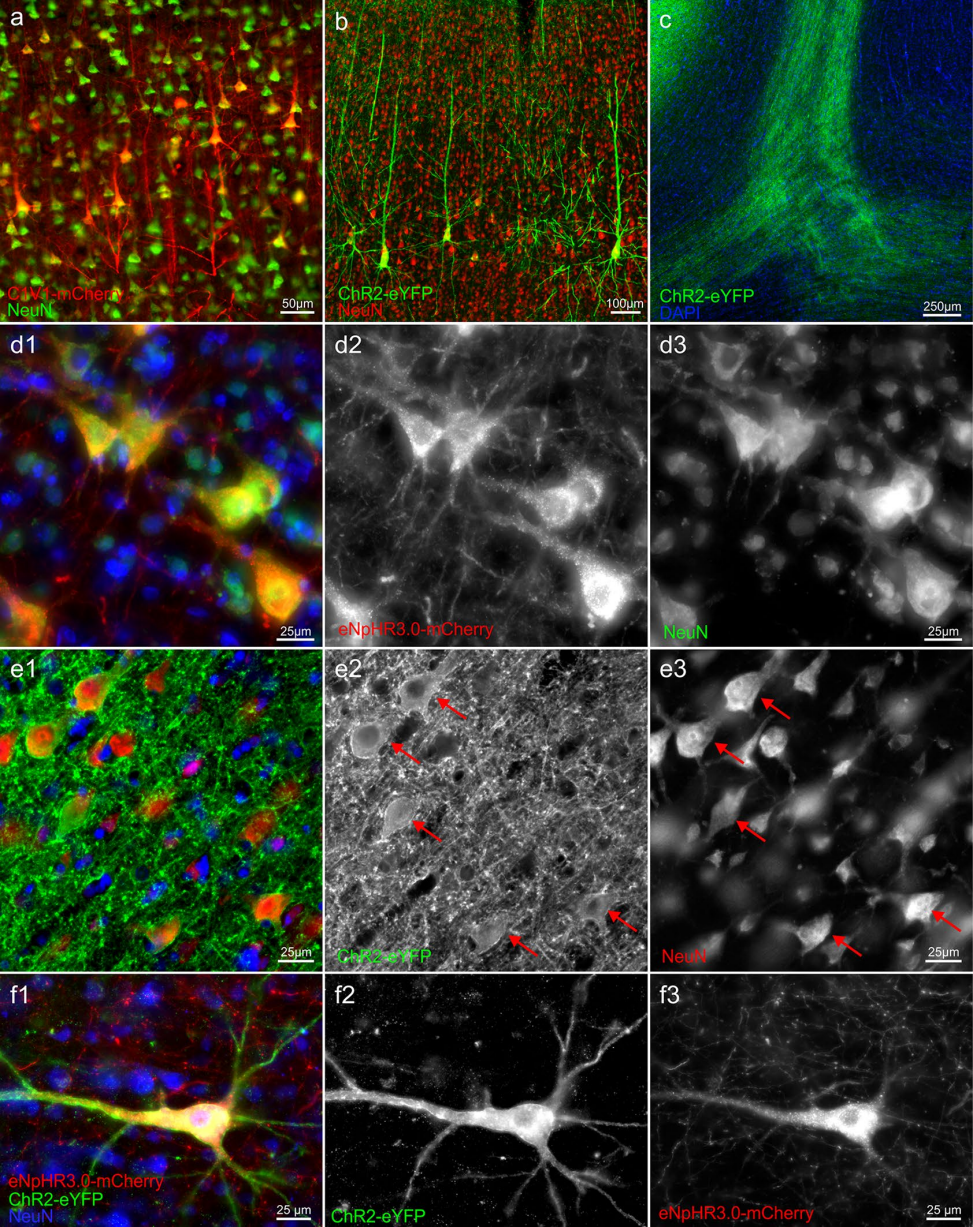
Opsin expression in cortical neurons. Morphology of typical cells expressing (**a**) C1V1-mCherry (pseudocolored in red) or (**b**) ChR2-eYFP (pseudocolored in green) and co-expressing NeuN (note reversed color scheme). (**c**) A bundle of ChR2-eYFP positive axonal fibers (green) in white matter, which originated from PMd neuron shown in Fig. 3b2 (area of magnification indicated by the box); section counterstained with DAPI. (**d1**) Higher magnification image illustrating eNpHR3.0-mCherry transduced (red, an original signal in (**d2**)) pyramidal neurons (NeuN positive—green, an original signal in (**d3**)) in area FEF in monkey O at the margins of the injection area; counterstained with DAPI (blue). Note punctated, vesicular-like eNpHR3.0-mCherry signal in transduced cell bodies. (**e1**) High magnification of ChR2-eYFP (green, an original signal in (**e2**)) transduced neurons (NeuN positive—red; an original signal seen in (**e3**)) in area PMd, counterstained with DAPI (blue). Cell bodies (arrows) were surrounded by dense fluorescent neuropil, making their unambiguous identification challenging. (**f1**–**f3**) Double-retrogradely transduced neuron found in area 7 m in monkey O; note the different cellular distribution of ChR2-eYFP (green) vs. eNpHR3.0-mCherry (red); counterstained with DAPI. Images acquired with Axio Observer 7 controlled by ZEN 2.3 (Zeiss).

We next analyzed in more detail the identity of opsin expressing cells. A vast majority of opsin-expressing cells, regardless of the construct used, displayed typical pyramidal neuron morphology: a conically shaped soma, a large apical dendrite projecting to upper cortical layers, and multiple basal dendrites (Fig. 5a,b). These morphological characteristics were further supported by the co-labeling with the neuron-specific nuclear protein (NeuN) (Figs. 4, 5). Cells that resemble glia were not observed. Most of the opsin-expressing neurons were found in putative layers V/VI and layers II/III, with an apparent lower density of transduced cells in granular layer IV and layer I (Fig. 4).

Cells expressing eNpHR3.0-mCherry and ChR2-eYFP also exhibited long axons to other cortical and subcortical areas (Figs. 2a2–b2, 3b2, 5c), including fibers crossing the midline and innervating the contralateral hemisphere (data not shown), which is another feature of excitatory pyramidal cells. In the case of the C1V1-mCherry opsin, this was not evident (e.g., compare b with c in Fig. 3), presumably due to weaker transgene expression or inefficient transport into axons and distal dendritic processes (see below).

**Figure 6:**
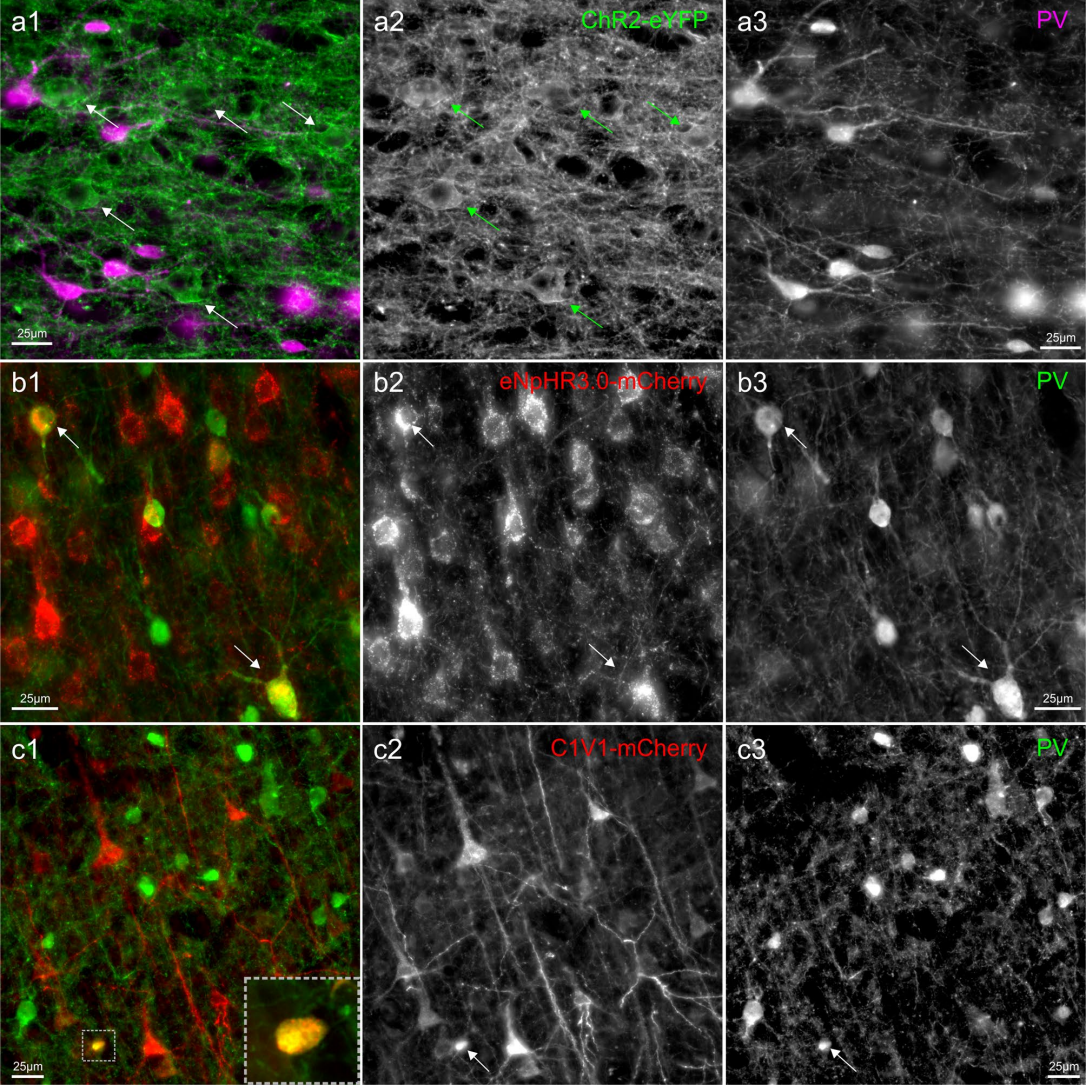
The specificity of CaMKIIα promoter-driven expression in cortical neurons. Immunostaining for parvalbumin (PV, a marker of mostly inhibitory neurons) along with (**a**) ChR2-eYFP, (**b**) eNpHR3.0-mCherry, and (**c**) C1V1-mCherry. In (**a1**), PV pseudocolored with magenta, in (**b1**) and (**c1**) pseudo-colored with red; an original immunofluorescent signal is shown in panels 2 and 3. Occasional PV^+^ cells could be seen in area FEF area co-expressing eNpHR3.0-mCherry transgene ((**b**), indicated with arrows), and to a lower degree in PMd of C1V1-mCherry injected area (**c**). A small, double-labeled cell shown in the inset in (**c1**); A very intense neuropil fluorescence in ChR2-eYFP injected areas (**a**) did not allow unequivocal determination, yet double-labeled cells were not noticed. Images acquired with Axio Observer 7, controlled by ZEN 2.3 (Zeiss).

To further investigate whether the CaMKIIα promoter restricts expression to excitatory neurons, we performed co-labeling with parvalbumin marker, which labels the largest subpopulation of GABAergic interneurons^28–30^ (Fig. 6). The great majority of transgene expressing neurons did not co-localize with PV^+^ cells. However, occasional double-labeled neurons could be observed (Fig. 6b,c). These cells accounted for a small fraction of all labeled neurons and were mostly encountered closer to the assumed center of the injection site. For C1V1-mCherry only ~ 1% of mCherry^+^ neurons were positively identified as also PV expressing (16 out of 1811 mCherry^+^ cells; 1,480 PV^+^ cells counted in the same area). In the case of FEF and eNpHR3.0-mCherry, the occurrence of double-labeled mCherry^+^/PV^+^ neurons was ~ 4.5% (88 out of 1970 mCherry^+^ cells; 1,029 PV^+^ cells counted in the same area), with a higher proportion of these cells seen close to the injection sites. Due to very intense neuropil fluorescence and poor discrimination of the cell bodies at the center of expressing areas (Fig. 6a), this quantification could not reliably be done for ChR2-eYFP.

Finally, as indicated earlier, we noticed different patterns of opsin expression within the cells when comparing all three opsin constructs. While ChR2-eYFP occurred almost exclusively on the plasma membrane, there was an additional, intense, and punctuated labeling of eNpHR3.0-mCherry inside the cell (with perinuclear enhancement) in what appeared to be vesicular compartments (Figs. 5d,e, 6a,b). This difference between the two constructs can be best observed in a double-labeled neuron shown in Fig. 5f. The C1V1-mCherry expression, instead, falls somewhat in between these two patterns, with the intracellular accumulation not as pronounced as in the case of eNpHR3.0-mCherry, and the membrane expression not as intense as for ChR2-eYFP (Figs. 5a, 6c).

### Opsin expression in projection areas

**Figure 7:**
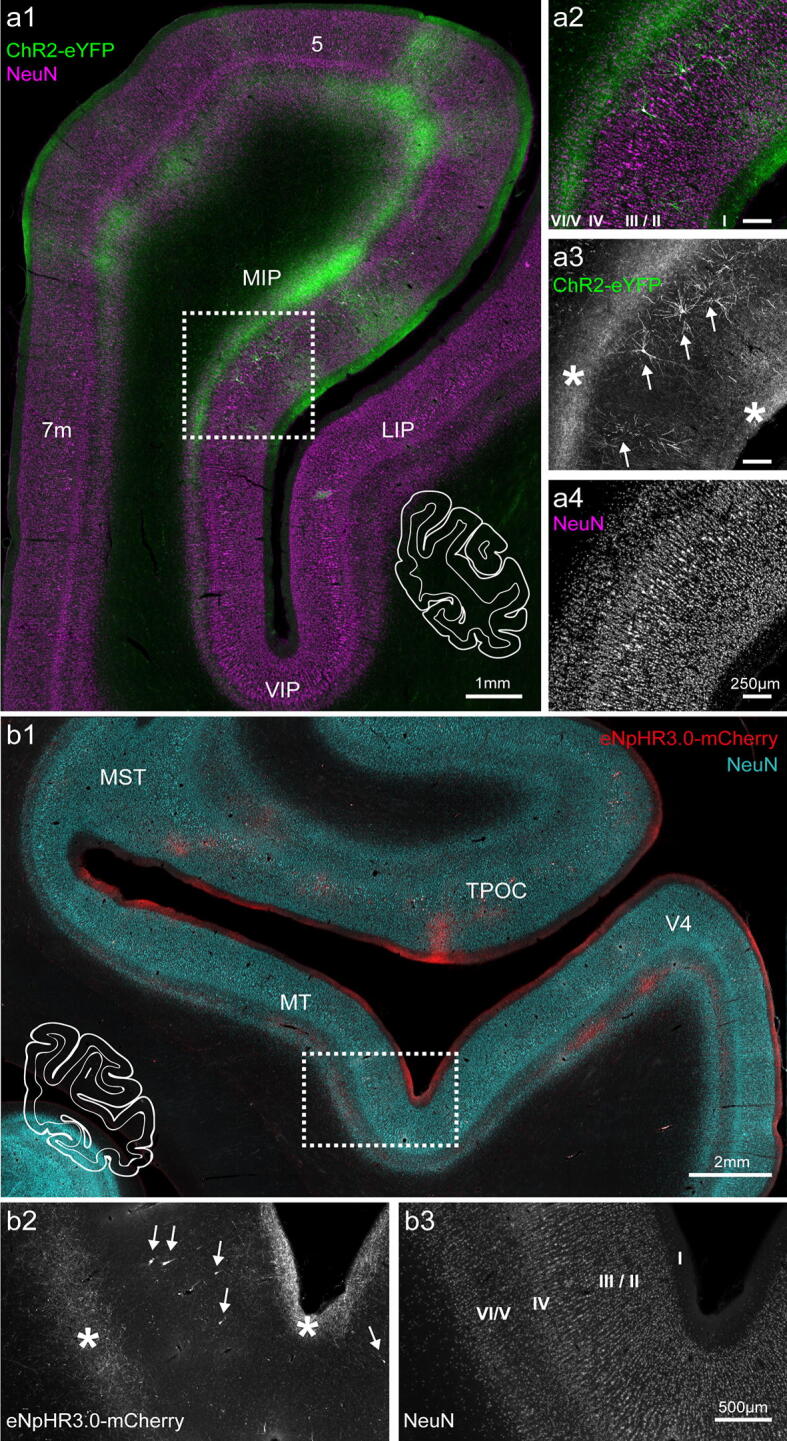
Axonal projections to the parietal lobe in monkey O. (**a**) Axonal projections from neurons in area PMd terminating in area MIP and area 5. Sections were immunoreacted against eYFP (green) and NeuN (magenta); (**a**) corresponding, white-contoured coronal hemisection is shown as a reference. (**a2**–**a4**) Magnification of the boxed area indicated in (**a1**). Projections were mainly found in layers V/VI, and to a lesser extent in layer I (asterisks in (**a3**)). A few retrogradely transduced neurons were present in deep layer III. (**b**) Axonal projections to area MT/MST; the outline of the complete coronal hemisection is shown for reference. Sections were immunoreacted against mCherry (cyan) and NeuN (red). (**b2**, **b3**) Magnification of the boxed area in (**b1**). Projections were found in layer I and layer V/VI (asterisks). Retrogradely transduced neurons could be found in layer II/III (indicated with arrows in (**b2**)). Images acquired with Axio Observer 7 controlled by ZEN 2.3 (Zeiss). 5, superior parietal lobule area; 7 m, medial parietal area; MIP, medial intraparietal area; LIP, lateral intraparietal area; VIP, ventral intraparietal area; TPOC, temporal parietooccipital associated area; V4, visual area 4.

We next examined how well the tested opsins were trafficked over long distances within the axons, given our overreaching aim to light-stimulate axonal projections in behaving NHPs. We examined the posterior parietal cortex (PPC; including medial intraparietal area: MIP, and lateral intraparietal area: LIP) and extrastriate visual areas (medial temporal area: MT, medial superior temporal area: MST), which are at a substantial distance, and are known to receive input from the injected frontal regions (PMd → PPC^31–33^, FEF → LIP^34^, and FEF → MT/MST^34,35^). In the case of ChR2-eYFP and eNpHR3.0-mCherry, axonal fibers could be easily identified in the target parietal and temporal areas (Fig. 7). Intense labeling of axons and terminals was observed predominately in the infragranular layers of the cortex, namely, layer V and VI, and also in layer I. This provides clear evidence for axonal membrane expression and efficient trafficking to distant brain regions (including brainstem and pons; data not shown) for ChR2 and eNpHR3.0. However, we were not able to detect (with our standard immunohistological procedure) axonal fibers originating from the C1V1-mCherry transduced cells – neither axonal projections nor retrogradely transduced cells could be found in expected locations in the parietal cortex or contralateral PMd (data not shown).

**Figure 8:**
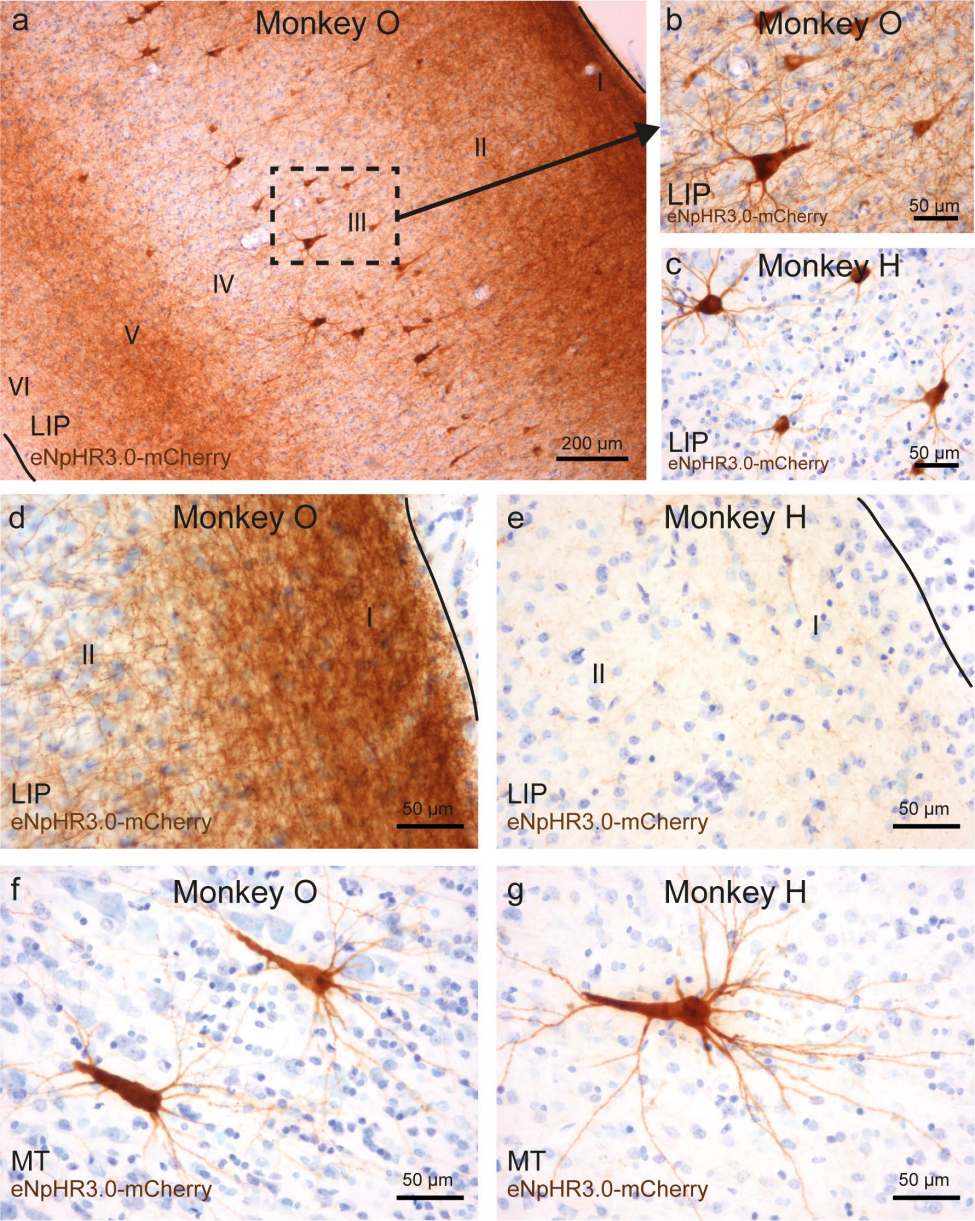
Cellular localization of opsins in projection areas at different time points post-injection. (**a**) Retrogradely transduced neurons in layer III, as well as axons and dendrites in all cortical layers show high expression of opsin constructs in monkey O. A direct comparison of area LIP in animals O (8.5 weeks post-injection) and H (2 years post-injection) shows opsin expression in all parts of retrogradely transduced neurons, as well as potentials axonal projections from FEF in monkey O (**a**,**b**), but mainly in the cell body and dendrites in monkey H (**c**). Opsins were present in axonal projections to layer I of LIP in monkey O (**d**) but could be barely detected in layer I of LIP in monkey H (**e**). Retrogradely transduced neurons in layer III of area MT showed a similar distribution of opsins within cell bodies and dendrites in monkey O (**f**) and monkey H (**g**). Images were captured with a BX51 Olympus light microscope, controlled by Cell^B software, version 3.4 (Olympus).

Projection target areas showed not only labeled processes but also individual or small groups of transduced neurons, mainly in supragranular layers predominantly putative layer III pyramidal cells (Fig. 7a,b). These retrogradely transduced perikarya, similarly to neurons at the injection site, displayed strong opsin expression in processes, with apical dendrites branching in upper cortical layers, resulting in additional local labeling of layers II/I, e.g., in superior parietal lobule area 5, MIP or in area MT (Fig. 7). The density of retrogradely labeled cells in these areas was low and ranged from 0.52 to 1.62 cells per mm^2^. In some parts of LIP, the number of eNpHR3.0^+^ retrogradely transduced neurons was somewhat higher (3.3 and 4.8 cells per mm^2^ in monkey H and O, respectively) and resulted in stained neuropil throughout all cortical layers (Fig. 8a). Supplementary Table S2 summarizes all individual counts from three animals.

Since we found retrogradely transduced neurons in distant cortical areas of the fronto-visual and fronto-parietal network, we also examined subcortical regions that contribute to corresponding transthalamic pathways and structures known to substantially project to our injection areas FEF and PMd^36–38^. For FEF injections of eNpHR3.0^+^, we assessed the superior colliculus, caudate nucleus, the mediodorsal (MD), ventrolateral (VL), and ventroanterior (VA) thalamus. Cell densities in the caudate nucleus (1.3 cells/mm^2^) and superior colliculus (1.8 cells/mm^2^) ranged between the values we found for MT and LIP. Cell densities in the MD and VL thalamus were higher (MD: 9.3., VL: 3.0 cells/mm^2^) and highest (30.8 cells/mm^2^) in AV thalamus (Supplementary Table S2). For PMd injections of ChR2, we did not find any relevant retrograde labeling in the examined subcortical structures.

### Comparison of opsin expression after different time periods

We compared the expression of eNpHR3.0-mCherry at two different time points: in monkey O at 8.5 weeks and in monkey H 2 years after virus injection. At the cellular level, opsin proteins were found in all parts of the neuron, i.e., cell bodies, dendrites, and axons within the injection area and corresponding white matter (Fig. 2). However, as indicated earlier, there was an apparent difference in the overall extent (and appearance) of expression in FEF between the two animals (see Fig. 2a, b). Even though monkey H received a larger virus injection, which was distributed over a bigger tissue volume than in monkey O, the transduction spread was similar in both animals. The tissue in monkey H also appeared less homogenously transduced, and some neuronal loss was evident (Supplementary Fig. S1). When comparing the projections in areas LIP and MT between the two animals, a considerable difference in the intensity of opsin-labeled axons could be seen; in monkey O, eNpHR3.0-mCherry protein density was high in the axons in both areas (Fig. 8a,b,d), while in monkey H, the labeling of axons in the projection areas was substantially weaker and more difficult to detect (Fig. 8c,e). This difference was most pronounced in long processes as the cell bodies and proximal dendrites of retrogradely transduced neurons did not show any obvious differences in the level of expression (Fig. 8b,c,f,g).

## Discussion

The aim of this study was to evaluate three commonly used optogenetic constructs in rhesus macaques for targeting cerebrocortical neural networks underlying visually guided behaviors and attention. As viral transduction methods are optimized and very effectively utilized in rodent models, they do not directly translate to primates^10,11,39^; hence, we tested an injection protocol intended to provide area-specific and homogenous expression spanning the thickness of the cortex within those networks. The same serotype AAV vectors were injected into areas FEF and PMd, and the resulting local expression and opsin localization in long-range axonal projections to parietal and visual areas were studied.

We report that injection into both areas resulted in robust expression of all three opsin constructs (ChR2-eYFP, eNpHR3.0-mCherry, C1V1-mCherry) at the injection sites. The CaMKIIα promoter provided strong neuronal expression, which was predominantly constrained to excitatory pyramidal cells, as less than 5% (eNpHR3.0) or as few as 1% (C1V1) of the opsin expressing cells were also parvalbumin-positive. We further show that ChR2-eYFP and eNpHR3.0-mCherry were efficiently trafficked in axons to distant projection areas, and thus can be used for projection-specific optogenetic manipulations, whereas C1V1-mCherry protein failed to meet this criterion. Finally, the tested opsin constructs displayed different cellular expression: ChR2-eYFP had the best membrane localization (including soma, dendrites, and axons), eNpHR3.0-mCherry showed intracellular accumulation additional to membrane expression, whereas C1V1-mCherry protein expressed in the soma and proximal dendrite membranes.

A few studies have successfully applied optogenetics in NHPs, also in areas FEF and PMd in NHPs^16–18,40–42^. However, many attempts to virally deliver opsin genes in NHPs proved ineffective and were regrettably not published (personal communication). We report that delivering 3–5 μl of a high titer virus, evenly divided along the cortical depth (1–1.5 μl depositions spaced by 1 mm), resulted in a transduction volume of 1–1.5 mm in radius, centered at the injection sites. Consequently, spacing individual penetration by ~ 2–4 mm (depending on the geometry of the area, and desired transduction density) should result in a fairly homogenous expression spread over a cortical region. Thus, the injection protocol reported here appears suited for transfecting large brain areas in rhesus monkeys, without the need of injecting massive volumes, as in the case of convection-enhanced delivery^43^.

Previous studies in NHPs^44,45^ indicated that the CaMKIIα promoter drives expression only in excitatory neurons, but not GABAergic neurons. In our analysis, however, the expression was not limited exclusively to excitatory neurons. Ectopic expression was infrequently seen in area FEF for eNpHR3.0-mCherry (cells were found more often close to the injection sites, deep in the tissue), and even to a lesser extent for C1V1-mCherry in area PMd, as the affected neurons marked a small fraction (~ 4.5% and 1% respectively) of opsin-positive neurons. A study in rodents suggests that within the context of AAV-mediated gene transfer, the CaMKIIa promoter is not entirely specific for glutamatergic neurons^46^. These observations are supported by studies in non-human primates^20,47–49^. Gerits and colleagues compared opsin expression using two different lengths of the CaMKIIα promotor (CaMKII0.4 and CaMKII1.3) and found that with the CaMKII1.3 promoter, 3% of transduced neurons were also PV-positive^47^. A study by Fetsch et al. reported that 9% of opsin-expressing neurons were PV-positive after injection into LIP^49^. It has also been shown in rodents, and in primates, that some (*potentially fast-spiking*) cortical pyramidal neurons can, in fact, express calcium-binding proteins like parvalbumin^50–55^. Thus, we can only speculate whether the opsin expressing PV^+^ neurons we found were partly pyramidal neurons and partly inhibitory neurons, since many of the double-labeled cells were rather small and did not display a typical pyramidal morphology. Therefore, the potential ectopic expression should be kept in mind when interpreting the data, or when one attempts to exclusively stimulate excitatory neurons.

For two of the opsin constructs tested (ChR2-EYFP and eNpHR3.0-mCherry), cellular expression was high in all functional compartments of neurons, i.e. soma, axons, and dendrites. FEF neurons projecting to LIP and MT, and PMd neurons projecting to area MIP displayed intense axonal labeling in projection areas, as well as in axon fibers crossing the midline, 2–4 months after viral vector injection; indicating that these two proteins are efficiently incorporated in the axonal membranes and that the long-distance projections can be optogenetically manipulated.

However, the C1V1-mCherry opsin protein was neither found in axonal projection to the parietal cortex nor in axons crossing the midline, and the expression seemed to be limited to cell bodies and proximal processes. This observation stands in contrast to two recent studies that reported expression of C1V1-eYFP in rhesus macaques in axonal fibers in subcortical white matter and axons projecting from the motor or premotor cortex to thalamus^8,42^. In both cases, similarly to our study, opsin genes were delivered with an AAV5 virus and under the CaMKIIα promotor, and the only apparent difference in the construct is the fluorescent reporter protein. Thus, this points to the possibility that specific opsin-reporter protein combinations might have a major effect on protein trafficking and the overall efficiency of an optogenetic approach. Indeed, we observed better membrane expression in the case of ChR2-eYFP, but quite obvious intracellular accumulations for both constructs carrying the mCherry gene (eNpHR3.0-mCherry and C1V1-mCherry).

Such intracellular accumulation, in turn, might have a negative effect on the overall health of cells in the long run. In this regard, the animal which expressed eNpHR3.0-mCherry for 2 years, showed some evidence of neuronal loss and changes in tissue integrity at the injection site (Supplementary Fig. S1). In addition, the density of the opsin protein (eNpHR3.0-mCherry) in axon terminals (i.e., in area LIP or MT) was much weaker than in a monkey with a shorter expression period. We suspect that the reported cell loss and tissue abnormalities are the results of an immune response to the long-term expression of eNpHR3.0-mCherry. We base this interpretation on the observation that the cell loss occurred predominantly around blood vessels and that histopathological staining by hematoxylin and eosin revealed perivascular cuffing and infiltration of the tissue by immune cells (Supplementary Fig. S1).

For circuit-level interpretations of optogenetic results, the possibility of retrograde uptake of the viral vector is an important consideration. We found cells in cortical and subcortical areas distant from the injection areas, which must have taken up the virus retrogradely via their axons. Previous optogenetic studies in macaques reported no or only small numbers of such cells after injection of AAV5^7,42,47^. However, a recent study found retrogradely transduced neurons in several brain areas after injection of AAV5 in the LGN, including the retina^48^. This is in line with systematic investigations in rodents, which showed the potential of AAV5 for retrograde transport^56,57^. After injection of the eNpHR3.0-mCherry construct into FEF, in some brain regions (e.g., area LIP, MD and VA thalamus), the number of retrogradely transduced neurons in our data was higher than in other regions (e.g., area MT, caudate nucleus), but overall still rather low with only few cells per mm^2^. Variations in density might reflect the strength of connectivity between FEF and these areas, or might be a cell-specific phenomenon, resulting from the presence of AAV-receptors on the axonal membrane of a given cell type. In any case, experiments with the aim to stimulate only the axon terminals in a target area should consider the possibility of retrograde uptake. After injection of the ChR2-eYFP construct in PMd, dense axonal projections were found in the thalamus or caudate nucleus, but no relevant retrograde uptake; rare uptake (less than 1–2 cells per mm^2^) was only found in cortical areas.

We have good reason to believe that the expression level of all three opsin constructs is sufficient to manipulate the activity of transduced neurons, although we did not test it systematically in all three animals. One of the animals (monkey H) was part of a neurophysiological study and showed clear changes in neuronal activity with laser stimulation, which we could also confirm in a second animal injected with the same viral construct in the same area (not part of this study).

In summary, our results indicate that optogenetics can be used to target the fronto-visual and fronto-parietal cerebrocortical networks in rhesus monkeys. Our injection protocol allows localized transduction of larger volumes of tissue, as required by the large primate brain. Optical stimulation experiments can be conducted locally within the injection area with all three constructs. Stimulation of axonal projections, even at distant target areas, should be possible with ChR2 and eNpHR3.0 within several months after injection. However, a decrease of opsin expression with time should be considered, and if histological verification cannot be afforded, interpretation of electrophysiological studies should account for the potential of stimulating a certain fraction of retrogradely transduced neurons.

## Methods

### Animals

Research with non-human primates represents a small but indispensable component of neuroscience research. The scientists in this study are aware and are committed to the responsibility they have in ensuring the best possible science with the least possible harm to the animals^39^. Details about our animal care and handling have been reported previously^58–60^. We summarize relevant details here:

All animal work was conducted according to the relevant national and international guidelines and in accordance with German laws governing animal use. All animal procedures have been approved by the responsible regional government office (Niedersächsisches Landesamt für Verbraucherschutz und Lebensmittelsicherheit [LAVES], Oldenburg, Germany) under the permit number 3392 42502–04-13/1100 as well as by the Animal Care Committee of the German Primate Center. All invasive procedures were done under appropriate anesthesia and with appropriate peri-surgical analgesia.

The experiments reported herein were performed on three adult male rhesus macaques (monkey O—11 years; monkey H—19 years; monkey G—8 years).

The animals were group-housed with other macaque monkeys in facilities of the German Primate Center in Goettingen, Germany in accordance with all applicable German and European regulations. The facility provides the animals with an enriched environment, including a multitude of toys, wooden structures and other enrichment^61,62^; as well as natural and artificial light, and exceeds the size requirements of the European regulations, including access to outdoor space. The German Primate Center has several staff veterinarians that monitor and examine the animals and consult on procedures. Throughout the study the animals' psychological and medical welfare was monitored by the veterinarians, the animal facility staff and the lab’s scientists, all specialized in working with non-human primates.

### Viral vectors and injection protocol

Viral vectors were injected during surgery in an operating room under general anesthesia. We determined the stereotactic coordinates of the injection sites based on prior anatomical MRI scans. A craniotomy was performed over PMd (monkey G, left hemisphere), area FEF (monkey H, left hemisphere), both (monkey O, right hemisphere). Each virus solution was loaded into a gas-sterilized Hamilton syringe (700 series; 10 μl or 25 μl volume, 32G needle with a sharp tip) under sterile conditions. For monkey H, the syringe was fixed to a manual injector, which was attached via a one-axis coarse manipulator (BE-8, Narishige) to a stereotaxic frame. For injections in monkeys G and O, a motorized integrated Stereotaxic injector (iSi, Stoelting) was used. After removing the bone, the dura was opened, and the locations for each needle penetration were determined based on stereotactic coordinates and visible anatomical landmarks (arcuate sulcus, spur of the arcuate sulcus and superior precentral dimple).

In all animals, injections were made at several neighboring locations—3 separate needle penetrations for area PMd, and 2 or 4 for area FEF). Along each penetration, a viral solution was delivered at multiple cortical depths and each injection point was separated by 1 mm in depth (see Fig. 1 with table for details). Virus injection started at the deepest point of each penetration, and 1 μl or 1.5 μl was injected at each location with a constant speed of 200 nl/min for 1 μl injections and 250 nl/min for 1.5 μl deposits. Each volume infusion was followed by a pause of 3 or 5 min, after which the needle was retracted by 1 mm to the next cortical depth; this procedure was then repeated for all locations.

Three different viral vectors were used in this study: (a) rAAV2/5-CaMKIIα-hChR2(H134R)-eYFP-WPRE (UNC Vector Core, titer: 8.5 × 10^12^ vg/ml), (b) rAAV2/5-CaMKIIα-eNpHR3.0-mCherry-WPRE (UNC Vector Core, titer: 4.7 × 10^12^ vg/ml), and (c) rAAV2/5-CaMKIIα-C1V1(E122T/E162T)-TS-mCherry (UNC Vector Core, titer: 5.4 × 10^12^ vg/ml).

Monkey G was injected with 12 µl of AAV2/5-CaMKIIα-C1V1-mCherry virus solution distributed evenly over three separate penetrations and forming an isosceles triangle (5 mm base and 3 mm height) in the caudal aspect of the premotor cortex (PMdc) and with 10.5 µl of AAV2/5-CaMKIIα-hChR2-eYFP virus targeted into rostral aspect of the premotor cortex (PMdr), also spread over three neighboring locations (4 mm apart—base, and 2 mm height—apex of an isosceles triangle) (see Fig. 1 for illustration of all the cases).

Monkey O was injected with 9 µl of AAV2/5-CaMKIIα-hChR2-eYFP virus into PMd in three separate penetrations (3 mm base, 2 mm height), and with 10 µl of AAV2/5-CaMKIIα-eNpHR3.0-mCherry virus delivered in FEF in two separate penetrations (distance between locations 2 mm).

Monkey H was injected with 16 µl of an AAV2/5-CaMKIIα-eNpHR3.0-mCherry in FEF distributed across four penetrations with a distance of 1.5–2 mm between them.

After completing injections, the brain surface was rinsed with sterile saline, and the dura was sutured. In monkey H and O, DuraGen (Integra, USA) was applied to the surface of the dura, and the craniotomy was covered with the previously removed piece of a bone. The bone was fixed to the surrounding skull via titanium strips and bone screws, the skin closed and sutured. In monkey G, the injection procedure was followed by implantation of a custom-made cranial chamber.

After the surgery, animals were placed in individual cages adjacent to their group housing rooms for post-surgery monitoring and returned to their social group after two days of recovery.

### Tissue fixation and processing

After variable survival periods (O—8.5 weeks, G—19 weeks, H—2 years,), the animals were euthanized with an intravenous injection of pentobarbital (1.5 ml/kg Narcoren, Boehringer Ingelheim Vetmedica GmbH), and, after cessation of all reflexes, transcardially perfused with heparinized phosphate-buffered saline (PBS), followed by 4% paraformaldehyde in PBS (PFA). After the perfusion, the calvarium was carefully removed and the head was placed in a stereotactic apparatus for dividing the brain into blocks of 9 – 15 mm at defined anterior–posterior (AP) levels. Brain blocks were post-fixed overnight by immersion in 4% (PFA), and then transferred into 10%, 20%, and 30% (w/v) sucrose in PBS, respectively, until sinking. Each block was adhered with Tissue-Tek O.C.T. Compound (Sakura Finetek) to an aluminum plate before rapid freezing in a metal bowl swimming on liquid nitrogen. A cryostat (Microm HM 500 OMV) was used to cut serial coronal sections of 50 µm, which were stored in PBS with 0.05% sodium azide at 4 °C for further processing.

### Immunofluorescence

All staining procedures were performed on free-floating sections. In short, sections were first washed once with PB (0.1 M phosphate buffer, pH 7.4), followed by three washes in TBS (Tris-buffered saline, pH 7.6), blocked with 10% normal serum (donkey + horse 1:1 or donkey only) in TBS with 0.2% Triton-X (TBS-T) for 1 h at RT and incubated with a primary antibody overnight in TBS-T with 2% serum (donkey + horse 1:1 or donkey only) at 4 °C. Following primary antibodies were used: (1) anti-GFP/EYFP (1:1,000, chicken, GFP-1020 from Aves labs), (2) anti-mCherry (1:500–1:1,000, rabbit, 600-401-P16 from Rockland) or Living Colors DsRed Polyclonal Antibody (rabbit, Catalog No. 632496 from Takara Bio USA, Inc.), (3) anti-NeuN (1:1,000, mouse, MAB377 from Millipore), and (4) anti-Parvalbumin (1:5,000, mouse, PV235 from Swant Inc.). Subsequently, sections were washed three times with TBS and incubated with a secondary antibody for 1–3 h at RT in TBS-T with 2% serum (donkey + horse 1:1 or donkey only). Secondary antibodies that were used: (1) Alexa 647-labeled donkey anti-mouse (1:400, code: 715-606-150), (2) Alexa 488 donkey anti-chicken (1:400, code: 703-546-155), (3) Cy3 donkey anti-rabbit (1:400, code: 711-166-152), or (4) Alexa 488 donkey anti-mouse (1:400, code: 715-546-150). All secondary antibodies and serums were acquired from Jackson ImmunoResearch Laboratories, Inc. (via Dianova). After three washes with TBS and three with PB, sections were mounted on glass slides, air-dried and coverslipped with a mounting medium (Fluoroshield Mounting Medium, Abcam, or Fluoromount-G with or without DAPI, Invitrogen).

### DAB-immunohistochemistry

All staining procedures were performed on free-floating sections. Sections were washed three times with PBS and incubated in 1% H_2_O_2_ in PBS for 30 min. They were again washed three times in PBS, followed by blocking in normal donkey serum (5% donkey serum in PBS + 0.3% Triton-X). They were incubated with a primary antibody (Rabbit Anti-mCherry, code: 600-401-P16, Rockland, dilution 1:1,000 or 1:500 in PBS with 1% donkey serum and 0.3% Triton-X) overnight at 4 °C. The sections were washed three times in PBS and then incubated with the secondary antibody for 1 h (Polyclonal Goat Anti-Rabbit biotinylated, code: E0432, Dako Denmark; dilution 1:400 in PBS with 1% donkey serum and 0.3% Triton-X). After incubation, sections were washed three times in PBS, after which the ABC peroxidase staining kit was applied (Thermo Scientific) for 1 h. The sections were washed three times with PBS, followed by DAB for 2 min, followed by three washes in PBS. They were mounted on gelatine-coated glass slides and dried at 37 °C overnight.

For counterstaining with cresyl violet, sections were rehydrated by passage through descending ethanol concentrations of ethanol (100%, 100%, 90%, 80%, 70%, 50%, Aqua bidest., each 10 s), followed by 0.3% cresyl violet for 3 s. The sections were then dehydrated by passing through an ascending ethanol series (50%, 70%, 80%, 90%, 100%, 100%, each 10 s), immersed in fresh Xylol for 2 × 3 min, and preserved under coverslips with Eukitt mounting medium.

### Imaging and quantification of transduction spread

To identify injection sites, every tenth serial coronal section of 50 µm (corresponding to 0.5 mm distance between consecutive sections) per brain block was used for each immunostaining, respectively, and imaged. All fluorescent images were taken with an Axio Observer 7 epifluorescent microscope (5 ×, 10 × and 25 × lenses, Zeiss, Jena, Germany) controlled by ZEN 2.3 (blue edition, Zeiss) and further processed with ImageJ (for fluorescent signal, standard image adjustments, including pseudo-coloring, z-stacks processing, threshold or brightness adjustments, were applied equally across the entire image).

DAB-stained slides were scanned with the Aperio CS2 scanner at a magnification of 20 × or 40 × (Leica, Germany), and the digitized images were analyzed with Aperio ImageScope (Version 12.3.2.5030, Leica Biosystems). Additional histological documentation (Fig. 8) was made by a BX51 Olympus light microscope with a Color View I Camera, controlled by Cell^B software, version 3.4 (Olympus).

To assess the effective spread of opsin expression, identified transduced areas were outlined and measured. The measurements from the cortical area PMd (ChR2-eYFP and C1V1-mCherry injections) are derived from images acquired by the epifluorescence microscope, whereas transduction spread in cortical area FEF was measured based on DAB stained sections (plus three additional immunofluorescent images to test for consistency between both methods). In both cases, the homogenous immunoreactive region around the potential needle tracks with clearly identifiable cell bodies at its margins and their immediate processes was outlined for each section (by the same observer, applying the same criteria), and the surface area and width of the outlined region quantified (built-in functions of ImageJ and Aperio ImageScope). Adjacent, irregular areas with immunoreactive processes, axons, and small clusters of putatively retrogradely labeled cells, which were clearly not part of the limited and homogenous injection sites, were not taken into account for this quantification.

### Quantification of retrogradely transduced neurons

Retrograde cells were counted in several cortical and subcortical areas (Supplementary Table S2). For each section, the anatomical structure was outlined, the surface area measured, and cells within this area counted (built-in functions of ImageJ and Aperio ImageScope). Cell density was computed as the number of neurons per area (mm^2^). This was repeated for all relevant sections (typically every tenth serial coronal section), and the resulting values averaged.

## Supplementary information

Supplementary file 1
