# Supplementary material for: Histological assessment of optogenetic tools to study fronto-visual and fronto-parietal cortical networks in the rhesus macaque
Source: Sci Rep. 2020 Jul 6;10:11051. doi: 10.1038/s41598-020-67752-6 (PMC7338380; doi:10.1038/s41598-020-67752-6)
Supplement: Supplementary file 1 [file 41598_2020_67752_MOESM1_ESM.pdf]

Histological assessment of optogenetic tools to study fronto-visual and fronto-parietal cortical networks in the rhesus macaque.

Michal G. Fortuna*†[1], Janina Hüer*†[1], Hao Guo[1], Jens Gruber[2], Eva Gruber-Dujardin[3], Jochen F. Staiger[4], Hansjörg Scherberger[5,6,7], Stefan Treue†[1,6,7], Alexander Gail†[1,6,7]

1 German Primate Center, Cognitive Neuroscience Laboratory, Göttingen, 37077, Germany
2 German Primate Center, Primate Genetics Laboratory, Göttingen, 37077, Germany
3 German Primate Center, Pathology Unit, Göttingen, 37077, Germany
4 University of Goettingen, University Medical Center, Institute for Neuroanatomy, Göttingen, 37075, Germany
5 German Primate Center, Neurobiology Laboratory, Göttingen, 37077, Germany
6 Bernstein Center for Computational Neuroscience, Göttingen, 37077, Germany
7 University of Goettingen, Faculty of Biology and Psychology, Göttingen, 37077, Germany

* corresponding authors: mfortuna@dpz.eu and jhueer@dpz.eu
† authors contributed equally to this work

Supplementary Figure S1

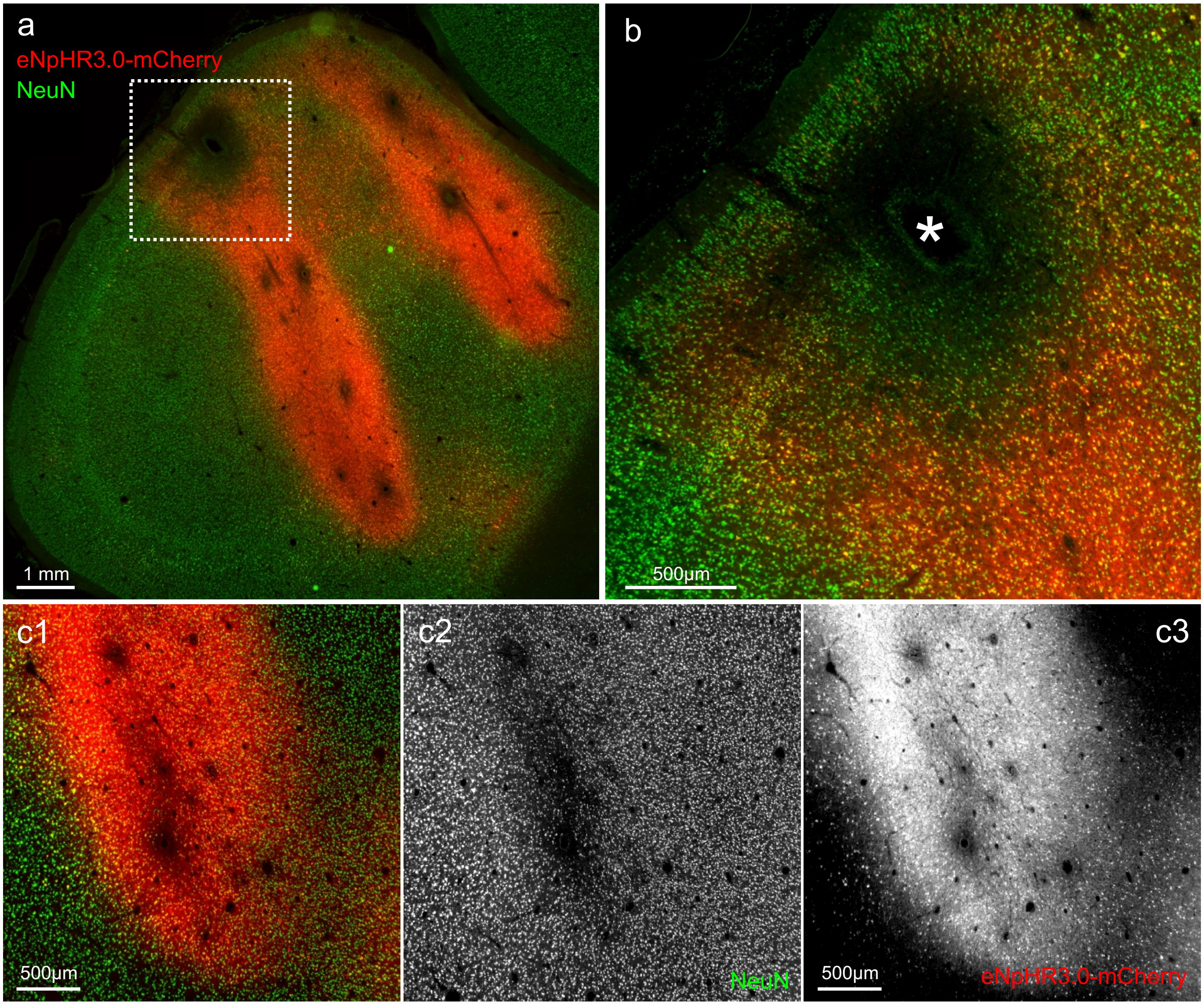

Supplementary Figure S1

Pathologies resulting from a long-term eNpHR3.0-mCherry expression in area FEF.

(**a**) A coronal section of the area FEF in monkey H, which was injected with the eNpHR3.0-mCherry virus, revealed with mCherry immunofluorescence (pseudocolored in red) and counterstained with NeuN (pseudocolored in green). Boxed area is shown in higher magnification in (**b**); note lower cell density due to potential cell loss around a putative blood vessel (marked with an asterisk).
In **c1-3**, eNpHR3.0-mCherry expression in FEF in the same animal but from a different anterior-posterior location (same section as shown in Fig. 2a). As in a/b, note locally lower cell density indicative of potential pathological changes; color scheme same as in a/b.

Supplementary Table S2

**Animal ID<br>Opsin<br>Area injected**	**Brain structure**	Cell count	# of section counted	Mean (± SE) cell count per structure	Mean (± SE) cell density per mm^2
**G**<br>hChR2-EYFP<br>**PMd**	***Posterior Parietal Cortex***				
	MIP	100	n = 9	10.2 ± 0.98	0.63 ± 0.06
	Area 5	53	n = 9	5.89 ± 1.15	0.52 ± 0.09
	Area 7m	161	n = 9	17.9 ± 1.39	0.97 ± 0.08
	Thalamus				
	VA + VL + MD	7	n = 7	1.0 ± 0.44	0.0
	RT	0	n = 7	0	0.0
	Caudate nucleus	1	n = 6	0.17 ± 0.17	0.0
**O**<br>hChR2-EYFP<br>**PMd**	***Posterior Parietal Cortex***				
	MIP	210	n = 8	26.2 ± 2.66	1.62 ± 0.12
	Area 5	80	n = 8	10.0 ± 1.67	0.76 ± 0.12
	Area 7m	51	n = 8	6.37 ± 0.99	0.38 ± 0.06
	Thalamus				
	VA + VL + MD	7	n = 6	1.17 ± 0.4	0.0
	RT	0	n = 6	0	0.0
	Caudate nucleus	0	n = 6	0	0.0
**O**<br>eNpHR3.0-mCherry<br>**FEF**	***Posterior Parietal Cortex***				
	LIP	615	n = 7	87.86 ± 7.35	4.85 ± 0.47
	Temporal area				
	MT	130	n = 7	18.57 ± 2.35	0.82 ± 0.09
	Thalamus				
	VA	237	n = 1	-	30.8
	MD	502	n = 3	167.3 ± 71.9	9.3 ± 4.73
	VL	330	n = 3	110 ± 51.1	3.0 ± 1.28
	Caudate nucleus	28	n = 2	14 ± 6	1.3 ± 0.08
**H**<br>eNpHR3.0-mCherry<br>**FEF**	***Posterior Parietal Cortex***				
	LIP	594	n = 7	84.86 ± 16.16	3.31 ± 0.59
	Temporal area				
	MT	79	n = 7	11.29 ± 1.94	0.48 ± 0.09
	Superior colliculus	34	n = 3	11.33 ± 0.33	1.85 ± 0.04

Supplementary Table S2

Rretrogradely labeled neurons in parietal and temporal target areas and selected subcortical structures.

A summary of cell count resulting from eNpHR3.0-mCherry virus injection in area FEF (monkey O and H) and hChR2-EYFP virus injection in area PMd (monkey G and O). Indicated brain structures (column 2) were identified in consecutive coronal sections (number of inspected sections specified in column 4), the area was measured, and cells within the area were counted. Column 3 provides the sum of cells found throughout the inspected area, and column 5 shows the average number of cells per each 50 μm section. Column 6 provides the average cell density for each area. Values are presented as mean ± standard error of the mean.
Abbreviations: MIP -medial intraparietal area, LIP – lateral intraparietal area, Area 5 - superior parietal lobule area, 7m – medial parietal area, MT - medial temporal area, VA -ventral anterior nucleus, MD - medial dorsal nucleus, VL - ventral lateral nucleus, RT - reticular nucleus.
